# The structural study of mutation-induced inactivation of human muscarinic receptor M4

**DOI:** 10.1107/S2052252520000597

**Published:** 2020-02-22

**Authors:** Jingjing Wang, Meng Wu, Lijie Wu, Yueming Xu, Fei Li, Yiran Wu, Petr Popov, Lin Wang, Fang Bai, Suwen Zhao, Zhi-Jie Liu, Tian Hua

**Affiliations:** a iHuman Institute, ShanghaiTech University, Shanghai 201210, People’s Republic of China; b School of Life Science and Technology, ShanghaiTech University, Shanghai 201210, People’s Republic of China; c CAS Center for Excellence in Molecular Cell Science, Shanghai Institute of Biochemistry and Cell Biology, Chinese Academy of Sciences, Shanghai, People’s Republic of China; d University of the Chinese Academy of Sciences, Beijing 100049, People’s Republic of China; e Skolkovo Institute of Science and Technology, Moscow, Russian Federation; f Shanghai Institute for Advance Immunochemical studies, ShanghaiTech University, Shanghai 201210, People’s Republic of China

**Keywords:** muscarinic acetylcholine receptors, G-protein-coupled receptors, M4, mutation design, ligand screening, Parkinson’s disease, Alzheimer’s disease, GPCRs

## Abstract

Human muscarinic receptor M4 belongs to the class A subfamily of the G-protein-coupled receptors (GPCRs). M4 has emerged as an attractive drug target for the treatment of Alzheimer’s disease and schizophrenia. Recent results showed that M4-mediated cholinergic transmission is related to motor symptoms in Parkinson’s disease. Selective ligand design for the five muscarinic acetylcholine receptor (mAchR) subtypes currently remains challenging owing to the high sequence and structural similarity of their orthosteric binding pockets. In order to obtain M4-selective antagonists, a new approach was tried to lock M4 into an inactive form by rationally designing an N449^7.49^R mutation, which mimics the allosteric sodium binding in the conserved sodium site usually found in class A GPCRs. In addition, the crystal structure of the mutation-induced inactive M4 was determined. By comparative analysis with other mAchR structures, followed by functional assays, the N449^7.49^R mutation was shown to stabilize M4 into an inactive state. Virtual screening of a focused ligand library using the crystal structure showed that the inactive M4 prefers antagonists much more than agonists. This study provides a powerful mutation strategy to stabilize GPCRs in inactive states and facilitate their structure determination.

## Abstract 2

A new approach of rationally designing an N449R mutation was developed to lock human muscarinic receptor M4 into an inactive form, and the crystal structure of the inactive M4 in a ligand-free form was determined.

## 1. Introduction   

Muscarinic acetylcholine receptors (mAchRs) are integral membrane proteins that belong to the class A G-protein-coupled receptors (GPCRs) and are activated by the neurotransmitter acetylcholine (Ach; Fredriksson *et al.*, 2003 ▸). Among the five muscarinic receptor subtypes, the M1, M3 and M5 subtypes couple to the G protein G_q/11_, activating phospholipase C and increasing cytosolic Ca^2+^, while the M2 and M4 subtypes couple to G_i/o_, mediating the inhibition of adenylyl cyclase (Hulme *et al.*, 1990 ▸). Each mAchR subtype also has a unique distribution throughout the peripheral or central nervous system in the human body, and they are attractive targets for the treatment of different pathophysiological conditions, including chronic obstructive pulmonary disease (COPD), overactive bladder and Sjögren’s syndrome (Eglen, 2012 ▸; Eglen *et al.*, 1996 ▸).

The high sequence and structural similarities among mAchR subtypes make selective ligand design quite challenging, which precludes the precise modulation of mAchR for therapeutic benefits. In the periphery, M4 is expressed in various pre-junctional nerve endings, acting to inhibit parasympathetic and sympathetic transmissions, while in the central nervous system (CNS) M4 is distributed in the corpus striatum and co-localizes with dopamine receptors on striatal projecting neurons. M4 also plays an important role in various motor disorder diseases such as Parkinson’s disease and dystonia (Bernard *et al.*, 1992 ▸; Eskow Jaunarajs *et al.*, 2015 ▸; Ztaou *et al.*, 2016 ▸). The majority of muscarinic antagonists, such as atropine and tiotropium, which is a commercial drug currently used for the treatment of COPD (Kato *et al.*, 2006 ▸), are nonselective. The weak M4-selective antagonist tropicamide is used to dilate the pupil for eye examinations or other diagnostic procedures (Lam *et al.*, 2010 ▸; Yazdani *et al.*, 2018 ▸). Several muscarinic acetylcholine receptor structures have been solved, as shown in Table 1 ▸. Most of them are in inactive states with nonselective antagonists or inverse agonists.

It is well established that the activation of class A GPCRs is initiated by ligand binding, which induces conformational changes of transmembrane (TM) helices. Upon activation, one of the most obvious features is that the cytoplasmic side of TM6 swings away from the transmembrane bundle. Several conserved or unique residues participate in the activation process. Firstly, the side chain of the toggle switch W^6.48^ (superscripts indicate Ballesteros–Weinstein numbering for GPCRs; Ballesteros & Weinstein, 1995 ▸) in TM6 undergoes a conformational change which unleashes a series of conformational movements of the receptor. Consequently, the residue at 3.46 breaks the contact with the residue at 6.37 and forms a new contact with the rotated Y^7.53^ within the highly conserved NP*xx*Y motif of TM7. The cytoplasmic ends of TM3 and TM6 are shown to disassociate owing to the breakage of a salt bridge between R^3.50^ at the end of TM3 (which is part of the conserved DRY motif) and E^6.30^ at the end of TM6.

High-resolution crystal structures have revealed that in­active class A GPCRs may harbour a conserved binding site for Na^+^ ions in the centre of their transmembrane domain (Fenalti *et al.*, 2014 ▸; Miller-Gallacher *et al.*, 2014 ▸; Zhang *et al.*, 2012 ▸). The Na^+^ ion is coordinated by a salt bridge to D^2.50^, together with four additional polar interactions with side chains of receptors and water molecules in the high-resolution crystal structures. For example, the Na^+^ ion in the human A_2A_ adenosine receptor (A_2A_AR) is coordinated by two highly conserved residues, D^2.50^ and S^3.39^, and three water molecules (Liu *et al.*, 2012 ▸). Na^+^ ions are found to selectively reduce the affinity of agonists but not antagonists, which is consistent with the structural stabilization of the inactive state by ions (Suno *et al.*, 2018 ▸). However, this Na^+^ ion-binding pocket is collapsed in active receptors. Mutations around the Na^+^ ion-binding site have a major impact on receptor function in most class A GPCRs, either completely abolishing G-protein coupling or resulting in constitutive ligand-independent or pathway-biased signalling (Suno *et al.*, 2018 ▸; Fenalti *et al.*, 2014 ▸; Huang *et al.*, 2015 ▸).

In order to identify potential new selective antagonists for M4, we set out to take a different approach by creating an inactive M4 induced by the rationally designed mutation N449^7.49^R, which is involved in the potential Na^+^-binding pocket in the transmembrane domains, to stabilize the protein. With five more mutations to aid in the expression and protein yield of M4, we further determined the crystal structure of the inactivated M4 receptor. Through comparative analysis of our crystal structure and tiotropium-bound M4 (PDB entry 5dsg; Thal *et al.*, 2016 ▸) and functional assays, the mutated M4 structure is shown to be in an inactive state. The virtual screening of a focused ligand library using our structure showed that antagonists are much more preferred than agonists, and that the mutation N449^7.49^R is the key element in preventing the activation of M4. Moreover, the inactivated mutation was so effective that a co-purifying, tightly bound ligand was trapped in the orthosteric site.

## 2. Experimental procedures   

### 2.1. Construction, protein expression and purification   

Wild-type M4 contains a long, probably poorly ordered third intracellular loop (ICL3) which is challenging for crystallization. To alleviate this problem, an M4-PGS (*Pyrococcus abyssi* glycogen synthase; PDB entry 2bfw; Horcajada *et al.*, 2006 ▸) fusion protein construct (Yin *et al.*, 2016 ▸) was generated using overlap PCR with six mutations: I93^2.65^T, G150^4.43^A, I187^ECL2^A, S219^5.62^Y, N449^7.49^R and T459^8.49^E. The construct was cloned into a modified pFastBac1 vector containing an N-terminal FLAG epitope tag followed by a 10×His tag. The construct included an HRV 3C cleavage site between S21 and S22 in the N-terminus. Residues 228–389 of ICL3 were replaced by PGS. The modified M4-PGS protein was expressed in *Spodoptera frugiperda* (*Sf*9) Super 3 insect cells using the Bac-to-Bac Baculovirus Expression System (Invitrogen). *Sf*9 Super 3 cells were infected at a cell density of 2–2.5 × 10^6^ cells per millitre with high-titre viral stock at a multiplicity of infection (MOI) of 5.0. Cells were harvested by centrifugation for 48 h post-infection and stored at −80°C for future use.

Frozen cell pellets were thawed and lysed by repeated washing and centrifugation in hypotonic buffer consisting of 10 m*M* HEPES pH 7.5, 10 m*M* MgCl_2_, 20 m*M* KCl and high osmotic buffer consisting of 10 m*M* HEPES pH 7.5, 10 m*M* MgCl_2_, 20 m*M* KCl, 1.0 *M* NaCl with EDTA-free cOmplete protease-inhibitor cocktail tablets (Roche). The washed membranes were suspended in hypotonic buffer with 30% glycerol, flash-frozen with liquid nitrogen and stored at −80°C until further use. Purified membranes were thawed at room temperature and incubated with 2.0 mg ml^−1^ iodoacetamide (Sigma) and inhibitor cocktail at 4°C for 1 h. The membranes were solubilized in a buffer consisting of 30 m*M* HEPES pH 7.5, 750 m*M* NaCl, 0.75%(*w*/*v*) lauryl maltose neopentyl glycol (LMNG; Anatrace) and 0.15%(*w*/*v*) cholesterol hemisuccinate (CHS; Sigma–Aldrich) at 4°C for 3 h. The supernatant containing the solubilized M4 proteins was isolated by high-speed centrifugation and then incubated with TALON IMAC resin (Clontech) and 20 m*M* imidazole at 4°C overnight. The resin was washed with 15 column volumes (CV) of washing buffer I consisting of 20 m*M* HEPES pH 7.5, 500 m*M* NaCl, 10%(*v*/*v*) glycerol, 0.03%(*w*/*v*) LMNG, 0.006%(*w*/*v*) CHS, 30 m*M* imidazole and 5 CV of washing buffer II consisting of 20 m*M* HEPES pH 7.5, 200 m*M* NaCl, 10%(*v*/*v*) glycerol, 0.01%(*w*/*v*) LMNG, 0.002%(*w*/*v*) CHS, 50 m*M* imidazole. The protein was eluted using 3 CV of elution buffer consisting of 20 m*M* HEPES pH 7.5, 150 m*M* NaCl, 10%(*v*/*v*) glycerol, 0.01%(*w*/*v*) LMNG, 0.002%(*w*/*v*) CHS, 250 m*M* imidazole. A PD MiniTrap G-25 column (GE Healthcare) was used to remove imidazole. The protein was treated overnight with HRV 3C protease to cleave the N-terminal FLAG/His tags from the protein. Finally, the purified protein was concentrated to about 50 mg ml^−1^ using a 100 kDa cutoff concentrator (Sartorius) and used in crystallization trials. The protein yield and monodispersity were tested by analytical size-exclusion chromatography. No ligands were added during the entire procedures.

### 2.2. Crystallization in lipidic cubic phase   

Crystallization was performed using the lipidic cubic phase (LCP) method (Caffrey & Cherezov, 2009 ▸). The concentrated M4-PGS was mixed with monoolein with 10%(*w*/*w*) cholesterol (Sigma) in a ratio of 2:3(*w*:*w*) using the syringe reconstitution method. The LCP mixture was dispensed in 35 nl droplets onto glass plates and overlaid with 800 nl precipitant solution using an NT8 robot (Formulatrix). The crystallization experiments were carried out in 96-well glass sandwich plates (Molecular Dimensions), which were subsequently stored in a Rock Imager (Formulatrix) at 20°C. Crystals of M4-PGS were obtained from precipitant conditions consisting of 300 m*M* diammonium hydrogen phosphate, 22–26% PEG 300, 0.1 *M* HEPES sodium pH 7.8 and reached a full size of 20–30 µm in 4–5 days, as shown in Supplementary Fig. S1.

### 2.3. Data collection and structure determination   

X-ray diffraction data were collected on beamline 41XU at SPring-8 using an EIGER X 16M detector (X-ray wavelength 1.0000 Å). Diffraction images were processed using *XDS* (Kabsch, 2010 ▸) and scaled with utilities from the *CCP*4 suite (Winn *et al.*, 2011 ▸). The structure was solved by molecular replacement with *Phaser* (McCoy *et al.*, 2007 ▸) using the M4–tiotropium structure (PDB entry 5dsg; Thal *et al.*, 2016 ▸) and the structure of the PGS domain (PDB entry 2bfw; Horcajada *et al.*, 2006 ▸) as separate models for the M4 and PGS fusion proteins. Refinement, rebuilding and structure determination were carried out using *Phenix* (Liebschner *et al.*, 2019 ▸), *BUSTER* (Smart *et al.*, 2012 ▸) and *Coot* (Emsley *et al.*, 2010 ▸). The structure was completed with *R*_work_ and *R*_free_ values of 0.231 and 0.264, respectively. The refinement statistics are summarized in Table 2 ▸.

### 2.4. Protein stability measurement using CPM assays   

Protein thermostability was measured using a microscale fluorescent thermostability assay as described previously (Alexandrov *et al.*, 2008 ▸). For the thermostability assay, CPM [7-diethylamino-3-(4′-maleimidylphenyl)-4-methylcoumarin] dye was dissolved in DMSO to 4 mg ml^−1^ as a stock solution and diluted 1:40 in buffer [20 m*M* HEPES pH 7.5, 200 m*M* NaCl, 10%(*v*/*v*) glycerol, 0.001%(*w*/*v*) LMNG, 0.0002%(*w*/*v*) CHS] before use. A total of 1 µl diluted CPM dye was added to the same buffer with approximately 0.5–2 µg M4 in a final volume of 50 µl. The samples were then incubated on ice for 15 min. The thermal denaturation assay was performed in a Rotor-Gene real-time PCR cycler (Qiagen). The excitation wavelength was 365 nm and the emission wavelength was 460 nm. All assays were performed over a temperature range from 25 to 99°C using a temperature rate of 2.0°C min^−1^. The stability data were processed with *GraphPad Prism*.

### 2.5. Split luciferase biosensor cAMP assay   

Human M4-expressing cell lines were generated using HEK293T cells. To validate the effects of the mutations on the M4 G_i_ protein signalling pathway, the split luciferase-based GloSensor cAMP biosensor technology (Promega) was employed followed by mutagenesis experiments. One day prior to assay, 4 µg M4 DNA and 5 µg GloSensor cAMP DNA (Promega) were co-transfected into HEK293T cells using Lipofectamine 2000 (Life Technologies). The cells grew for 5 h and half of the culture medium was replaced by equilibration medium. The cells were then added into 384-well white poly-d-lysine-coated plates (Greiner) with Dulbecco’s Modified Eagle Medium (DMEM; Life Technologies) supplemented with 1% dialyzed fetal bovine serum (dFBS) at a density of 15 000–20 000 cells in 40 µl medium per well and incubated overnight (20–24 h) at 37°C in 5% carbon dioxide. The following day, the culture medium was removed from the cell plates. The wells were loaded with 20 µl 2 mg ml^−1^ d-luciferin sodium salt prepared in Hanks’ balanced salt solution (HBSS) pH 7.4 and incubated for 1 h at 37°C. All of the following steps were carried out at room temperature. To measure the Ach activity at M4, 10 µl 3× Ach solution was added with a final concentration ranging from 1 n*M* to 3 µ*M* and reacted for 15 min. The plates for the agonist assay were diluted by adding 10 µl isoproterenol (Sigma) at a final concentration of 200 n*M*, paused for 15 min and followed by measuring the luminescence using an EnVision plate reader (Perkin Elmer). Data were analysed using *GraphPad Prism*.

### 2.6. Molecular docking   

Prediction of ligand binding to M4 was performed with *Schrödinger Suite* 2018-4 (Schrödinger). The protein structures were processed with the *Protein Preparation Wizard* and the structures of ligands were prepared by *LigPrep*. Molecular docking was carried out with *Glide*.

### 2.7. Molecular-dynamics simulation of M4 in complex with agonists and antagonists   

The processed crystal structure of M4 in complex with either an agonist or an antagonist was embedded in a bilayer composed of 140 1-palmitoyl-2-oleoyl-*sn*-glycero-3-phospho­choline (POPC) lipids using the *CHARMM-GUI Membrane Builder* (Jo *et al.*, 2008 ▸; Wang *et al.*, 2004 ▸). The protein orientation in the membrane is referenced to M4 (PDB entry 5dsg) in the Orientations of Proteins in Membranes (OPM) database (Lomize *et al.*, 2012 ▸). The protein–membrane system was solvated in a periodic 0.15 *M* NaCl TIP3P water box with a minimum water height of 20.0 Å at the top and bottom of the system. All simulations were performed on a GPU cluster using the CUDA version of *PMEMD* (particle mesh Ewald molecular dynamics) in *Amber*18 (University of California, San Francisco). The protein was modelled with the ff14SB protein force field (Maier *et al.*, 2015 ▸), ligands with the GAFF2 force field (Wang *et al.*, 2004 ▸) and lipids with the AMBER Lipid17 force field. The constructed system was first energy-minimized for 10 000 steps; the first 5000 of these were performed using the steepest-descent method and the remaining 5000 used the conjugate-gradient method. The simulation system was then heated from 0 to 100 K using Langevin dynamics with a constant box volume. Restraints were applied to protein, ligands and lipids with a constant force of 10 kcal mol Å^−2^. Subsequently, the temperature was increased to 310 K, where the periodic box was coupled accordingly using anisotropic Berendsen control in order to maintain the pressure at around 1 bar. The particle mesh Ewald (PME; Darden *et al.*, 1993 ▸) method was used to treat all electrostatic interactions beyond a cutoff of 9 Å. The *SHAKE* algorithm (Ryckaert *et al.*, 1977 ▸) was used to record the length of bonds involving hydrogen during the simulation, with an integration time step of 2 fs. Further equilibration was then carried out at 310 K with harmonic restraints applied to the protein starting at 5 kcal mol Å^−2^ and reduced in a stepwise fashion every 2 ns for 10 ns, followed by 0.1 kcal mol Å^−2^ restraints for 20 ns to give a total of 30 ns of equilibration. 2 µs production simulations with no restraints were then performed at 310 K and 1 bar in the NPT ensemble for each system for three independent runs, and these trajectories were used for analysis with *Visual Molecular Dynamics* (*VMD*; Humphrey *et al.*, 1996 ▸) and *CPPTRAJ* (Roe & Cheatham, 2013 ▸).

## 3. Results   

### 3.1. Rationally designed mutation-induced inactive M4   

**Figure 1:**
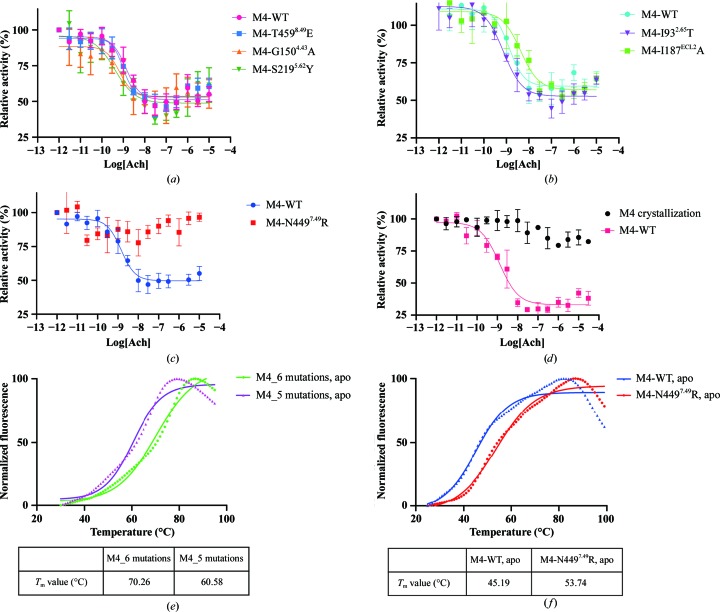
Pharmacological characterization and thermostability assay of mutants of M4. (*a*, *b*) Dose–response studies of agonist Ach activity for each mutant compared with wild-type M4 (M4-WT). The EC_50_ values (mean ± SEM) of Ach are 1.059 ± 0.1489, 1.036 ± 0.139, 1.777 ± 0.6448, 2.314 ± 1.012, 0.9343 ± 0.1192 and 6.357 ± 1.262 n*M* for the M4-WT, T459^8.49^E, G150^4.43^A, S219^5.62^Y, I93^2.65^T and I187^ECL2^A constructs, respectively. (*c*, *d*) G_i_ activation assays of M4 with the key point mutation N449^7.49^R and M4 with six mutations as a function of Ach compared with that of M4-WT. (*e*) Thermostability assay of the crystallization construct with six mutations (M4_6 mutations) and a construct with the other five mutations apart from N449^7.49^R (M4_5 mutations), where the fusion protein used in the constructs is PGS. (*f*) Thermostability assay of M4-N449^7.49^R and M4-WT with the fusion protein modified T4 lysozyme. The N449^7.49^R mutant showed an increase in the melting temperature (*T*_m_) by about 8.1 ± 1.1°C.

Point mutations have been shown to be an effective method to improve the expression and the thermostability of GPCRs (Peng *et al.*, 2018 ▸; Popov *et al.*, 2018 ▸, 2019 ▸). After rational design and intensive screening, six mutations were introduced to improve the expression of M4 (S219^5.62^Y, G150^4.43^A, T459^8.49^E, I187^ECL2^A, I93^2.65^T and N449^7.49^R). Among these, S219^5.62^Y, G150^4.43^A and T459^8.49^E increase the thermostability of M4, while I187^ECL2^A and I93^2.65^T have been reported to increase the binding affinity of ligands and the cooperativity of positive allosteric modulators (Chan *et al.*, 2008 ▸; Thal *et al.*, 2016 ▸). N449^7.49^R was rationally designed to play a similar role to the sodium ion in order to lock M4 into an inactive state. In the functional assay assessing the regulation of adenylyl cyclase, the first five mutations only showed a less than tenfold increased or decreased signalling activity compared with wild-type M4 receptor [Figs. 1 ▸(*a*) and 1 ▸(*b*)]. However, the N449^7.49^R mutation almost abolished the signalling and led to M4 that was completely inactive towards Ach, and the construct with these six mutations also showed no activity in the functional assay [Figs. 1 ▸(*c*) and 1 ▸(*d*)]. More importantly, the N449^7.49^R mutation significantly increased the thermostability of the receptor by 8°C compared with the construct without the N449^7.49^R mutation [Figs. 1 ▸(*e*) and 1 ▸(*f*)].

### 3.2. Crystal structure determination of the mutation-induced inactive M4   

To facilitate crystallization, the M4 sequence was further modified by inserting a soluble fusion protein within the third intracellular loop 3 (ICL3), which is long and poorly ordered. In this work, PGS (*P. abyssi* glycogen synthase) was identified as a stabilizing fusion partner. The M4-PGS construct was expressed in *Spodoptera frugiperda* (*Sf*9) Super 3 insect cells using the Bac-to-Bac Baculovirus Expression System (Invitrogen). The construct yielded about 1.5 mg of M4 protein per litre from *Sf*9 Super 3 cells. The protein yield was improved greatly with the key mutation N449^7.49^R. The M4 crystals were formed in lipidic cubic phase (Caffrey & Cherezov, 2009 ▸) and diffracted to 3.0 Å resolution, as shown in Table 2 ▸. Unexpectedly, unknown electron density was observed in the orthosteric binding pocket even though no ligands were added from cell growth to crystallization. The unknown density position also differs from that of PEG 300, which was present in the allosteric binding pocket in inactive M3 and M4 structures (Liu *et al.*, 2018 ▸; Thal *et al.*, 2016 ▸). As the purified protein showed a high *T*_m_ value without the addition of any ligands, we proposed that the unknown molecule may bind to the receptor during expression.

In order to identify the unknown binding molecule, we tried the molecular-docking method and virtually screened 114 157 metabolite entries from the Human Metabolome Database (HMDB, http://www.hmdb.ca/), which covers most of the eukaryotic cell metabolites. Three fatty acids (HMDB0010212, HMDB0034295 and HMDB0010217) showed relatively high potential for binding with M4. We then modelled the three fatty acids into the electron density and performed structure refinement. The results showed that all of them fit well into the density (Supplementary Fig. S4), and the *R*_work_ and *R*_free_ decreased after refinement (Supplementary Table S2).

In addition, previous studies have shown that high *n*-6 polyunsaturated fatty acid (PUFA) diets can influence the binding affinity of muscarinic receptors (Farkas *et al.*, 2002 ▸; Flynn & Mash, 1993 ▸; Freund *et al.*, 1986 ▸). The *n*-6 PUFA group displayed a reduction in M2/M4 binding in the caudate putamen, anterior cingulate cortex and hippocampus (du Bois *et al.*, 2005 ▸). The unknown density observed in this study may provide some evidence that M4 might potentially bind to endogenous polyunsaturated fatty acids.

### 3.3. Comparison with known mAchR structures   

**Figure 2:**
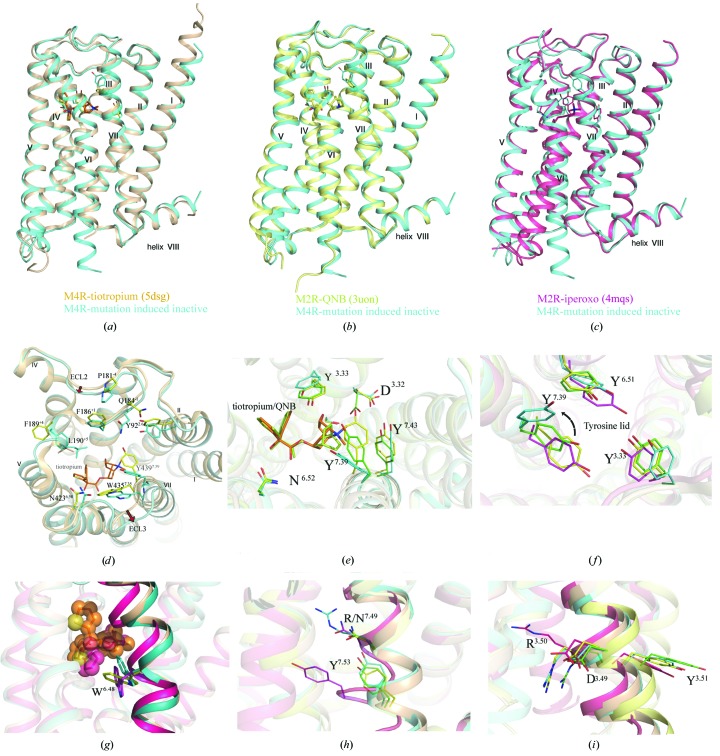
Comparison of the mutation-induced inactive M4 structure with structures of M2 and M4 in inactive and active states. (*a*, *b*, *c*) M2 and M4 are aligned with the mutation-induced inactive M4 structure (teal blue). The M4–tiotropium structure is shown as an orange cartoon and the inactive and active M2 structures are shown as pale yellow and magenta cartoons, respectively. (*d*) Extracellular region comparison of mutation-induced inactive M4 and M4–tiotropium (PDB entry 5dsg) structures. (*e*) The highly conserved residues in orthosteric binding pockets for tiotropium and QNB. (*f*) The ‘tyrosine lid’ is formed by three tyrosines: Y^6.51^, Y^3.33^ and Y^7.39^. There is a 110° rotation of Y^7.39^ compared with that in M4–tiotropium; the arrow shows the rotation of Y439^7.39^ in the mutation-induced inactive M4 structure. (*g*) The different rotations of W^6.48^ in the mutation-induced inactive M4, active M2 and M4–tiotropium structures. (*h*, *i*) Comparison of the NP*xx*Y and D(E)RY motifs between the inactive and active mAchR structures.

To date, crystal structures of M1, M3 and M4 bound to the antagonist tiotropium have been determined, as well as a QNB-bound M2 structure, as shown in Table 1 ▸. The ligands share almost identical binding poses in the orthosteric binding pockets [Fig. 2 ▸(*d*)]. The overall structure of mutation-induced inactive M4 is similar to the structures of antagonist-bound mAChRs, with an especially high similarity between TM3, TM6 and TM7. For example, the root-mean-square deviation (r.m.s.d.) between mutation-induced inactive M4 and the M4–titropium complex structure (PDB entry 5dsg) is only 0.5–0.7 Å (Supplementary Table S1). Compared with the solved inactive mAChR structures, the mutation-induced inactive M4 structure has a more open extracellular domain owing to the outward movement of the extracellular region of TM1, TM6, TM7 and ECL3 [Figs. 2 ▸(*a*) and 2 ▸(*b*)]. The electron-density maps for the seven transmembrane helices and typical key residues are shown in Supplementary Fig. S5.

The orthosteric binding pocket is mainly composed of the D112^3.32^, Y113^3.33,^ S116^3.36^, N417^6.52^ and Y439^7.39^ residues in mAChR structures. Compared with the M4–tiotropium complex structure, the side chains of these residues in the mutation-induced inactive M4 structure show outward movements. Additionally, the mutation-induced inactive M4 structure shows several unique features; for example, the side chain of Y439^7.39^ in the conserved tyrosine lid rotates about 110°, which forms the aromatic cage enclosing the amine and forms a lid over the orthosteric pocket [Fig. 2 ▸(*e*)]. With the rotation of Y439^7.39^, the network of hydrogen-bond inter­actions collapses, creating a largely hydrophobic binding cavity between the orthosteric and allosteric binding pockets. The N449^7.49^R substitution allowed an unknown hydrophobic ligand to be trapped in a hydrophobic pocket of the ortho­steric site, which further stabilized the inactive state. This rotation of Y439^7.39^ also occurs in the docking results for some other M4-selective agonists, in which Y439^7.39^ adopted an ‘open’ conformation in all selected templates, including the agonist iperoxo (Yang *et al.*, 2019 ▸), which differs from what was observed in the reported X-ray structure of the activated form of M2 (Kruse *et al.*, 2013 ▸). The conformations of the orthosteric and allosteric sites are coupled such that the presence of a ligand in one site affects the shape of the other (Burger *et al.*, 2018 ▸).

The side chain of W413^6.48^, the ‘toggle switch’ residue, rotates about 45° compared with the M4–tiotropium structure, but differs from that in the active M2 structure (PDB entry 4mqs; Kruse *et al.*, 2013 ▸) [Fig. 2 ▸(*f*)]. In addition, the salt bridge between D^3.49^ and R^3.50^ in the conserved D(E)RY motif is still intact, further indicating that the structure is in the inactive state [Figs. 2 ▸(*g*) and 2 ▸(*h*)].

### 3.4. The mechanism of mutation-induced inactivation of M4   

**Figure 3:**
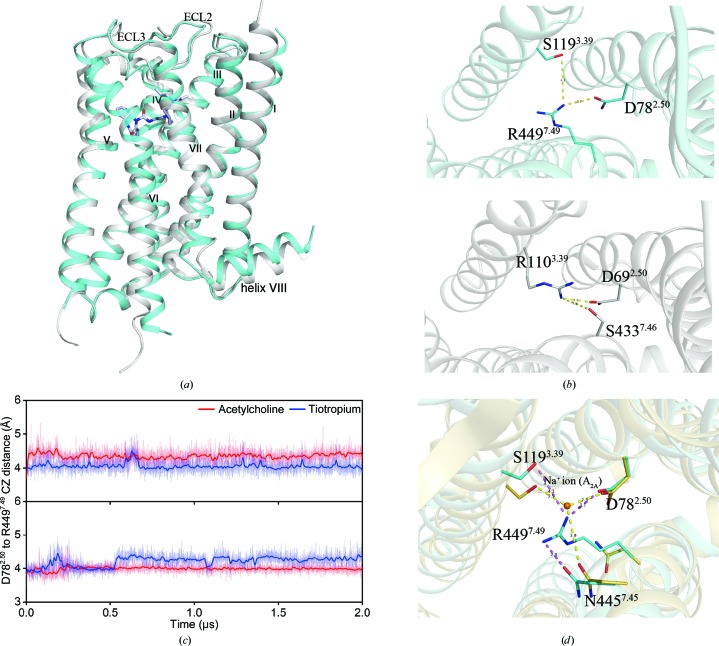
The ionic networks in the mutation-induced inactive M4 structure. (*a*) Alignment of the structures of M2–AF-DX 384 (PDB entry 5zkb; grey cartoon) and mutation-induced inactive M4 (teal blue cartoon). (*b*) In the mutation-induced inactive M4 structure (top), S119^3.39^, D78^2.50^ and R449^7.49^ form an ionic network, while in the M2 structure (bottom) D69^2.50^, S433^7.46^ and R110^3.39^ form a salt-bridge interaction. (*c*) The distance between CG of D78^2.50^ and CZ of R449^7.49^ during MD simulation of Ach-bound or tiotropium-bound M4_1_ (top) and M4_6_ (bottom). The detailed information is from the computational analysis of mutation-induced inactive M4. (*d*) Comparison of the sodium-binding site in the ZM241385–A_2A_ structure (PDB entry 4eiy; lemon) and the ionic network around R449^7.49^ in mutation-induced inactive M4.

D^2.50^ is highly conserved among the class A GPCRs and is important for coordinating the binding of the allosteric sodium ion in many cases (Vickery *et al.*, 2018 ▸; Katritch *et al.*, 2014 ▸). In the recent M2–AF-DX 384 structure, the point mutation S110^3.39^R was designed based on the structure of the A_2A_ adenosine receptor (PDB entry 4eiy; Liu *et al.*, 2012 ▸) and the S110^3.39^R mutation acts like the sodium cation and forms a salt bridge with D^2.50^ (Suno *et al.*, 2018 ▸) [Fig. 3 ▸(*b*)]. In our mutation-induced M4 structure, N449^7.49^R in the NP*xx*Y motif forms a salt bridge with D78^2.50^, as well as a hydrogen bond to S119^3.39^ [Fig. 3 ▸(*b*)]. The ionic network, especially the inter­action between D78^2.50^ and the mutated R449^7.49^, locks the M4 structure into an inactive state [Fig. 3 ▸(*d*)]. Notably, on comparison with the structure of the A_2A_ receptor with sodium ion (PDB code 4eiy), the positive charge of R449^7.49^ in the mutation-induced inactive M4 has the same function as the allosteric sodium ion in the A_2A_ structure [Fig. 3 ▸(*d*)]. In class A GPCRs, polar amino acids are present with a 98% probability at N^7.49^ of the NP*xx*Y motif, with a 78% probability of asparagine (Supplementary Fig. S2). The probabilities of serine at 3.39 and aspartic acid at 2.50 are 70% and 96%, respectively. Thus, the N^7.49^R mutation approach should have a high success rate in stabilizing most class A GPCRs in an inactive form to facilitate structure determination.

### 3.5. Computational analysis of mutation-induced inactive M4   

**Figure 4:**
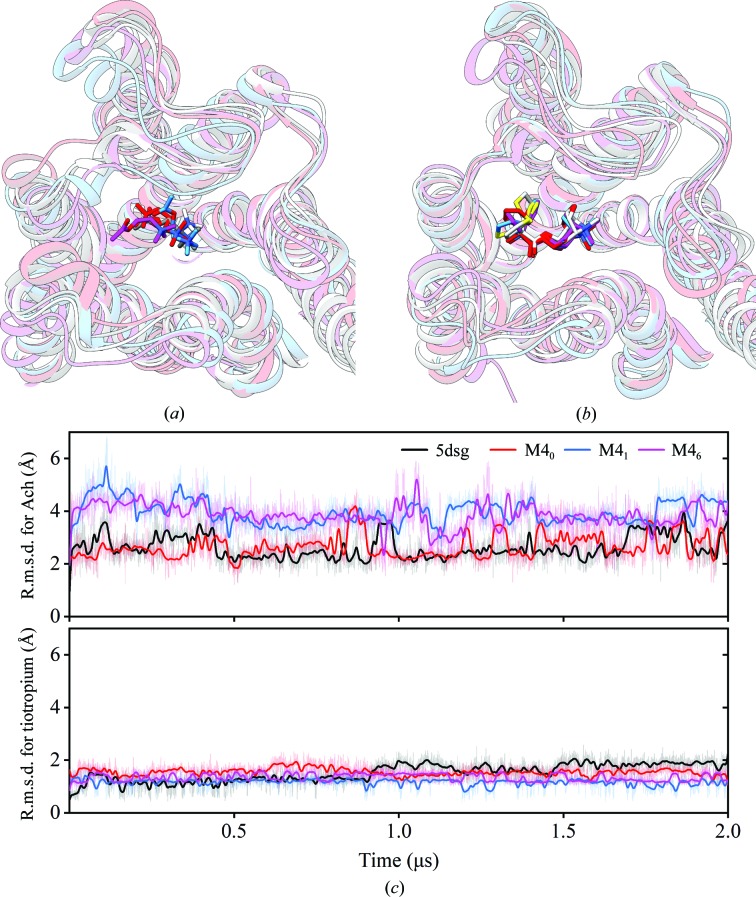
Molecular-dynamics simulations of different forms [M4–tiotropium (PDB entry 5dsg), M4_0_, M4_1_ and M4_6_] of M4. The last frames from the trajectories of the protein with Ach (*a*) and tiotropium (*b*) were aligned to show the locations of the ligands in M4_6_ (purple), M4_1_ (blue), M4_0_ (red) and M4–tiotropium (PDB entry 5dsg; dark colour). (*c*) R.m.s.d. of the agonist Ach (top) and the antagonist tiotropium (bottom) with respect to the protein and its binding pocket during the simulations. Tiotropium is stable in the M4_6_ and M4_1_ templates when compared with Ach in the binding pocket.

To verify whether the mutation-induced M4 structure could be used as an inactive template for *in silico* screening of potential antagonists, we performed molecular docking of known mAchR ligands using the new structure. The computational analysis was divided into two steps. In the first step, in order to further probe whether the N449^7.49^R mutation is the key factor that induces the M4 into an inactive state, docking and molecular-dynamics (MD) simulations were performed using four different forms of M4, as shown in Table 3 ▸: (i) the structure of M4 bound to the antagonist tiotropium (PDB entry 5dsg) as a positive control, (ii) M4_6_, the crystal structure of mutation-induced M4 (with six mutations) in this study, (iii) M4_1_, a modified M4 with the N449^7.49^R mutation only, and (iv) M4_0_, a modified M4 without any mutations. Ach and tiotropium were selected as a representative agonist and antagonist, respectively. Their initial docking poses were obtained by superimposing iperoxo-bound M2 (PDB entry 4mqs) or tiotropium-bound M4 (PDB entry 5dsg) onto the four M4 structures that were just described. The composed Ach-bound or tiotropium-bound M4 template structures were embedded in a lipid bilayer and each MD simulation system was fully equilibrated and relaxed; three independent 2 µs production simulations with no restraints were then performed at 310 K and 1 bar in a constant total number of particles, pressure and temperature (NPT) ensemble for each case (Table 3 ▸). After the MD runs, the final binding poses of Ach and tiotropium, including the M4 structures, are shown in Fig. 4 ▸. Not surprisingly, Ach has diffused away from its initial binding site and has conformationally fluctuated quite significantly in terms of r.m.s.d. values, while the r.m.s.d. values for the antagonist tiotropium were obviously lower. Remarkably, for M4_6_ and M4_1_, which contain the N449^7.49^R mutation, Ach is more mobile throughout the entire simulation compared with tiotropium-bound M4 (PDB entry 5dsg) and M4_0_. Accordingly, tiotropium is stable in M4_6_ and M4_1_ template structures, which is consistent with PDB entry 5dsg (Fig. 4 ▸). This further confirms that the M4_6_ structure is indeed in an inactive state and that its orthosteric pocket prefers to bind antagonists.

**Figure 5:**
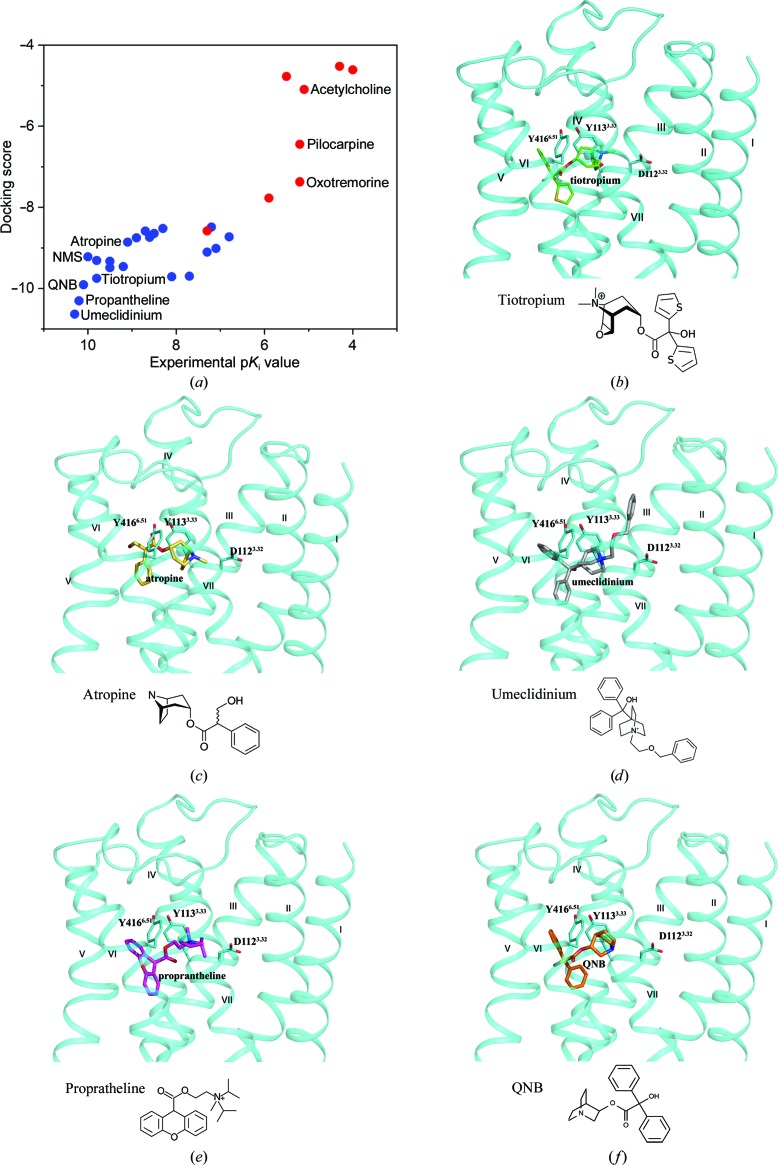
Molecular docking of muscarinic receptor ligands using the mutation-induced inactive M4 structure. (*a*) The results are listed with compounds in descending order of docking score along with the experimental p*K*_i_ value. Antagonists and agonists are shown as blue and red dots, respectively. (*b*–*f*) The chemical structures and predicted binding poses of antagonists obtaining high scores in (*a*). (*b*) Tiotropium (green sticks), (*c*) atropine (yellow sticks), (*d*) umeclidinium (grey sticks), (*e*) propantheline (purple sticks) and (*f*) QNB (orange sticks).

To further investigate the performance of the mutation-induced M4 structure in the virtual screening of potential antagonists with a broader ligand library, we carried out molecular docking against a focused muscarinic receptors compound library selected from the IUPHAR database (http://www.guidetopharmacology.org). The selected library is composed of 90 ligands, including agonists, antagonists and allosteric modulators for muscarinic receptors. All of the compounds in this library showed efficacies against certain muscarinic receptors in both functional and binding assays. The docking was performed with *Schrödinger Suite* 2018-4 (Schrödinger) and the results are listed in descending order by docking scores along with the experimental p*K*_i_ values (Fig. 5 ▸). In agreement with our results above, the top-ranked compounds in the list are antagonists. Five M4 antagonists, tiotropium, atropine, proprantheline, QNB and umeclidinium, were selected as representatives of different scaffolds for further analysis and their predicted binding poses are shown in Fig. 5 ▸. These five antagonists share similar binding poses, mainly interacting with D112^3.32^, Y113^3.33^ and Y416^6.51^.

## 4. Discussion   

Recent structural studies on muscarinic acetylcholine receptors provided important structural insights into the receptor–ligand interactions and ligand-binding poses in orthosteric or allosteric binding pockets. In this study, we demonstrate how to obtain a GPCR structure in its inactive state via key point mutations. In this specific case, we took M4 as an example and created a single point mutation, N449^7.49^R, to stabilize M4 in its inactive form. We further crystallized and determined the crystal structure of the mutation-induced inactive M4. In a structural comparison with agonist-bound and antagonist-bound mAchRs structures, the mutation-induced inactive M4 structure is obviously similar to the antagonist-bound M4 structure. However, our mutation-induced M4 trapped an unidentified, co-purifying ligand that bound to M4 like a high-affinity antagonist. In addition, we observed that the positively charged N449^7.49^R residue mimics the allosteric sodium ion binding to the conserved residues D78^2.50^ and S119^3.39^, which lock M4 into an inactive state. Also, the intracellular domains of the mutation-induced inactive M4 tend to fold into a tighter structural conformation compared with the M4–titropium structure by forming hydrophilic interactions among R130^3.50^, T399^6.34^, R144^ICL2^ and E395^6.30^. Taken together, the sodium-mimicking ionic lock between TM3 and TM6 and the ionic interaction between R130^3.50^ and E395^6.30^ stabilize M4 in an inactive state. The N449^7.49^R mutation and the switches of TM3 and TM6 were so effective in stabilizing the inactive state that a co-purifying hydrophobic molecule was trapped. Through further computational analysis using molecular docking and MD simulations of M4 structures of different forms, the N449^7.49^R mutation is shown to be the key mutation which makes M4 prefer to bind antagonists compared with agonists.

GPCRs are important drug targets and are involved in virtually every biological process. In this study, we experimentally and computationally validated that the mutation N449^7.49^R effectively inactivated M4. The designed ionic bond network is formed by S^3.39^, D^2.50^ and N^7.49^R, which is similar to the effect of the S^3.39^R mutation in mimicking allosteric Na^+^ binding in the conserved Na^+^ ion-binding site in class A GPCRs, as previously reported. Thus, either the N^7.49^R or S^3.39^R mutation may apply to other class A GPCRs to stabilize the receptor in an inactive state and to facilitate structure determination in complex with an antagonist or in an apo state.

## Supplementary Material

PDB reference: muscarinic receptor M4, 6kp6

Supplementary tables and figures. DOI: 10.1107/S2052252520000597/jt5041sup1.pdf

## Figures and Tables

**Table 1:** List of mAChRs with known structures

mAChR	Structure state	PDB code	Resolution (Å)	Ligand	Reference
M1	Inactive	5cxv	2.7	Tiotropium	Thal *et al.* (2016 ▸)
	Active	6oij	3.3	Iperoxo	Maeda *et al.* (2019 ▸)
M2	Inactive	3uon	3.0	QNB†	Haga *et al.* (2012 ▸)
		5zkc	2.3	NMS‡	Suno *et al.* (2018 ▸)
		5yc8	2.5	NMS	Suno *et al.* (2018 ▸)
		5zkb	2.95	AF-DX 384	Suno *et al.* (2018 ▸)
		5zk8	3.0	NMS	Suno *et al.* (2018 ▸)
		5zk3	2.6	QNB	Suno *et al.* (2018 ▸)
	Active	4mqt	3.7	Iperoxo, LY2119620	Kruse *et al.* (2013 ▸)
		4mqs	3.5	Iperoxo	Kruse *et al.* (2013 ▸)
		6oik	3.6	Iperoxo, LY2119620	Maeda *et al.* (2019 ▸)
M3	Inactive	4daj	3.4	Tiotropium	Kruse *et al.* (2012 ▸)
		4u14	3.57	Tiotropium	Kruse *et al.* (2012 ▸)
		4u15	2.8	Tiotropium	Kruse *et al.* (2012 ▸)
		4u16	3.7	NMS	Kruse *et al.* (2012 ▸)
		5zhp	3.1	6o(BS46)	Liu *et al.* (2018 ▸)
M4	Inactive	5dsg	2.6	Tiotropium	Thal *et al.* (2016 ▸)

† *R*-(2)-3-quinuclidinyl benzilate.

‡ *N*-Methylscopolamine.

**Table 2:** Data-collection and structure-refinement statistics for mutation-induced inactive M4 Values in parentheses are for the highest resolution shell.

	No ligand	HMDB0010212 docked
Data collection		
Wavelength (Å)	1.000	
Resolution range (Å)	50.00–3.00 (3.08–3.00)	
Space group	*P*2_1_2_1_2_1_	
*a* (Å)	56.10	
*b* (Å)	61.32	
*c* (Å)	203.74	
Observed reflections	398969	
Unique reflections	14718	
Multiplicity	27.1 (6.0)	
Completeness (%)	99.7 (97.0)	
Mean *I*/σ(*I*)	19.3 (2.3)	
Wilson *B* factor (Å^2^)	89.34	
*R*_merge_ †	0.132 (0.586)	
CC_1/2_ ‡	0.996 (0.379)	
Refinement		
Resolution range (Å)	49.14–3.00	
Reflections (work/test)	13985/731	
*R*_work_/*R*_free_ §	0.231/0.264	
No. of atoms		
Total	3711	3734
Macromolecules	3711	3711
Ligands	0	23
Solvent	0	0
No. of protein residues	474	477
R.m.s.d., bonds (Å)	0.008	0.014
R.m.s.d., angles (°)	0.94	1.67
Ramachandran statistics¶		
Favoured (%)	96.39	94.48
Allowed (%)	3.41	5.10
Outliers (%)	0.42††	0.42††
Clashscore	2.42	3.75
PDB code	6kp6	

† *R*_merge_ = 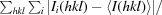 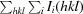, where *I_i_*(*hkl*) is the intensity of observation *i* of reflection *hkl*.

‡ As defined by Karplus & Diederichs (2012 ▸).

§ *R* = 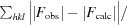 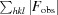 for all reflections, where *F*_obs_ and *F*_calc_ are the observed and calculated structure factors, respectively. *R*_free_ is calculated analogously for the test reflections, which were randomly selected and excluded from the refinement.

¶ As defined by *MolProbity* (Chen *et al.*, 2010 ▸).

†† Glycine residues 1075 and 1144 are in sharp-turn domains of the PGS fusion protein.

**Table 3:** Molecular-dynamics simulation of the agonist Ach and the antagonist tiotropium with different forms of M4

System	Mutation details	Ligand	Simulation time (µs) × No. of runs
PDB entry 5dsg	None	Ach	2 × 3
		Tiotropium	2 × 3
M4_0_	None	Ach	2 × 3
		Tiotropium	2 × 3
M4_1_	N449^7.49^R	Ach	2 × 3
		Tiotropium	2 × 3
M4_6_	I93^2.65^T, G150^4.43^A, I187^ECL2^A, S219^5.62^Y, N449^7.49^R, T459^8.49^E	Ach	2 × 3
		Tiotropium	2 × 3
