# Supplementary material for: The structural study of mutation-induced inactivation of human muscarinic receptor M4
Source: IUCrJ. 2020 Feb 22;7(Pt 2):294–305. doi: 10.1107/S2052252520000597 (PMC7055379; doi:10.1107/S2052252520000597)
Supplement: Supplementary file 1 [file m-07-00294-sup1.pdf]

IUCrJ

Volume 7 (2020)

Supporting information for article:

The structural study of mutation-induced inactivation of human muscarinic receptor M4

Jingjing Wang, Meng Wu, Lijie Wu, Yueming Xu, Fei Li, Yiran Wu, Petr Popov, Lin Wang, Fang Bai, Suwen Zhao, Zhi-Jie Liu and Tian Hua

Table S1 Supporting information RMSD values of the mutation-induced inactive M4 structure with other classical mAChRs structures

**State**	**mAchRs (PDB code)**	**ligand**	**RMSD value (Å)**
Inactive	M1R(5CXV)	tiotropium	1.227
	M2R(3UON)	QNB*	0.713
	M3R(4U15)	tiotropium	0.701
	M4R(5DSG)	tiotropium	0.699
Active	M1R(6OIJ)	iperoxo	1.472
	M2R(6OIK)	iperoxo	1.251

* R-(2)-3-quinuclidinyl benzilate.

Table S2 Supporting information R_{free}/R_{work} values of the mutation-induced inactive M4 structure with three fatty acids from docking results

HMDB ID	Compound name	Chemical structure	R_{free}/R_{work} (%)
None	None		26.42/23.14
0010212	17,18-EpETE	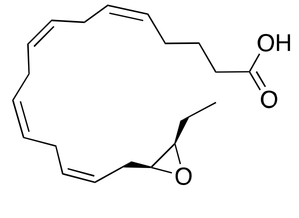	26.79/21.65
0034295	Floionolic acid	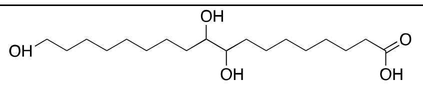	26.45/21.72
0010217	5-oxo-ETE	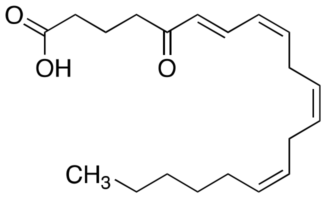	26.70/21.63

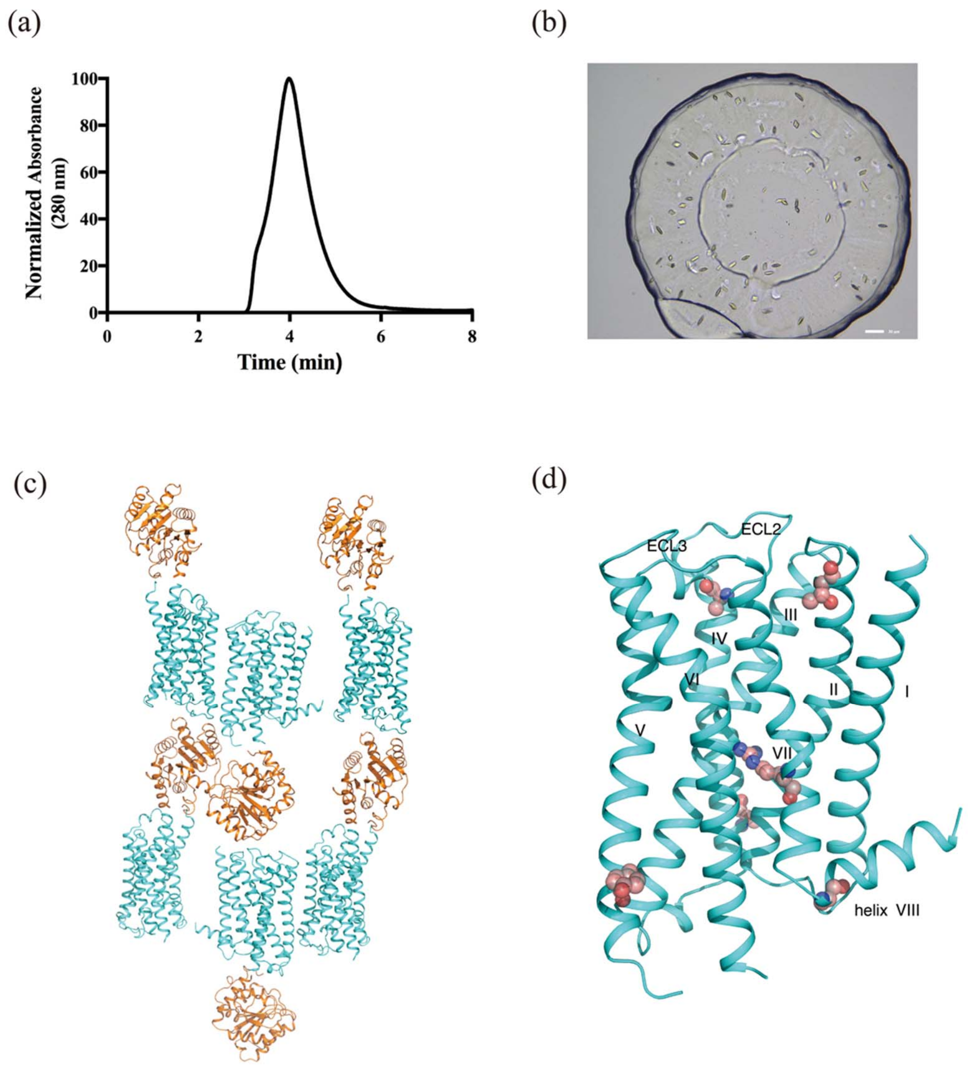

Figure S1 Protein purification and crystal packing of the mutation-induced inactive M4 structure. (a) Analytical size-exclusion chromatography trace of purified mutation-induced M4 protein. (b) Crystal picture of M4 obtained in lipidic cubic phase. (c-d) Crystal packing and the overall structure of mutation-induced inactive M4. M4 and PGS are coloured in teal blue and orange, respectively. Six-point mutations in the crystallization construct are shown as pink spheres.

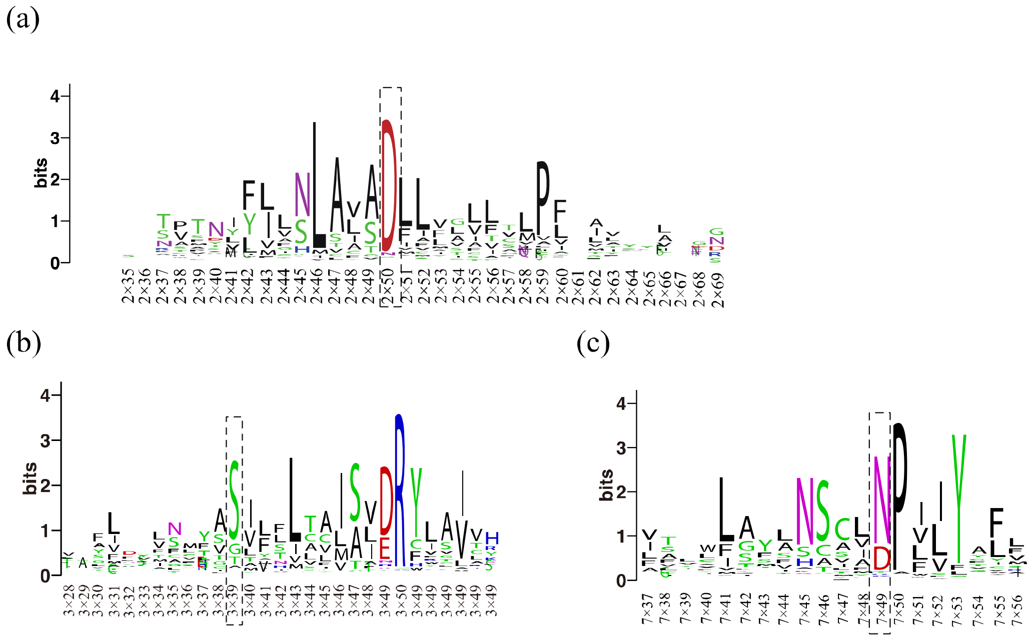

(d)

GPCRDB number	Polar Amino acids	Asn (N)	Asp (D)	Glu (E)	Gln (Q)	His (H)	Ser (S)	Thr (T)	Tyr (Y)
2×50	98%	1%	**96%**	1%	0%	0%	0%	0%	0%
3×39	78%	0%	0%	1%	0%	0%	**70%**	7%	0%
7×49	98%	**78%**	17%	0%	0%	1%	1%	1%	1%

Figure S2 Residues in the rational designed ionic network are conserved in class A GPCRs. (a-c) TM2, TM3 and TM7sequence conservation across 286 human class A GPCRs (including 81 orphan receptors and non-olfactory receptors). (d) The percentages of the polar amino acids in the conserved positions of 2×50 in TM2, 3×39 of TM3 and 7×49 in TM7.

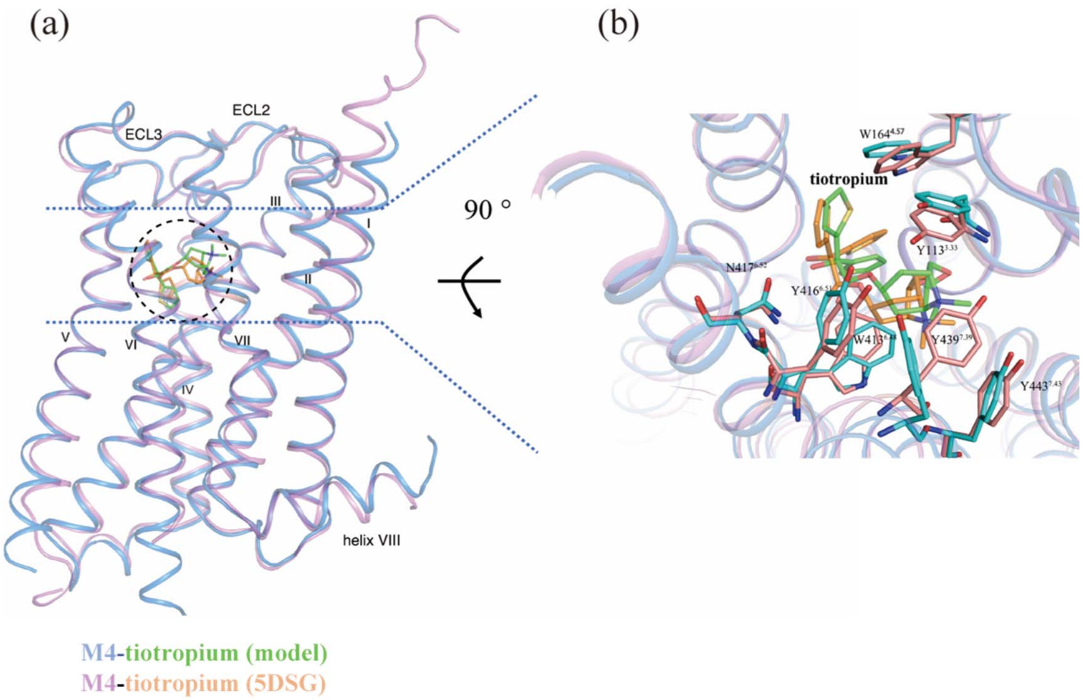

Figure S3 Molecular docking and molecular dynamic simulation results of tiotropium using the mutation-induced inactive M4 structure. (a) The overall comparison with M4-tiotropium structure (PDB code 5DSG). (b) The interaction residues in the orthosteric binding pocket are similar except for side chains of W164$^{4.57}$, Y416$^{6.51}$ and Y439$^{7.39}$.

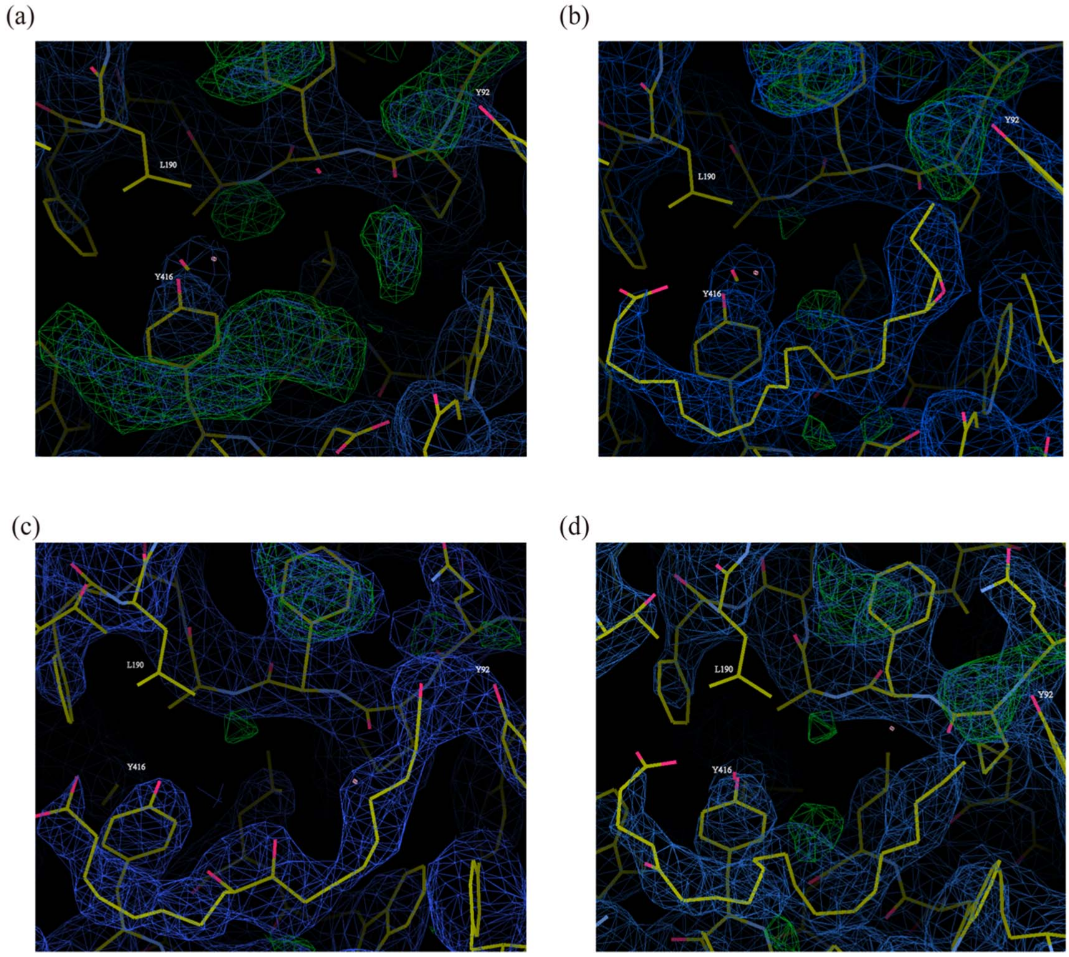

Figure S4 Electron density maps for three different fatty acids. (a) The initial omit 2|F_o|-|F_c| map. (b-d) The refined 2|F_o|-|F_c| maps for 17,18-EpETE (b), Floionolic acid (c) and 5-oxo-ETE (d), after refinements.

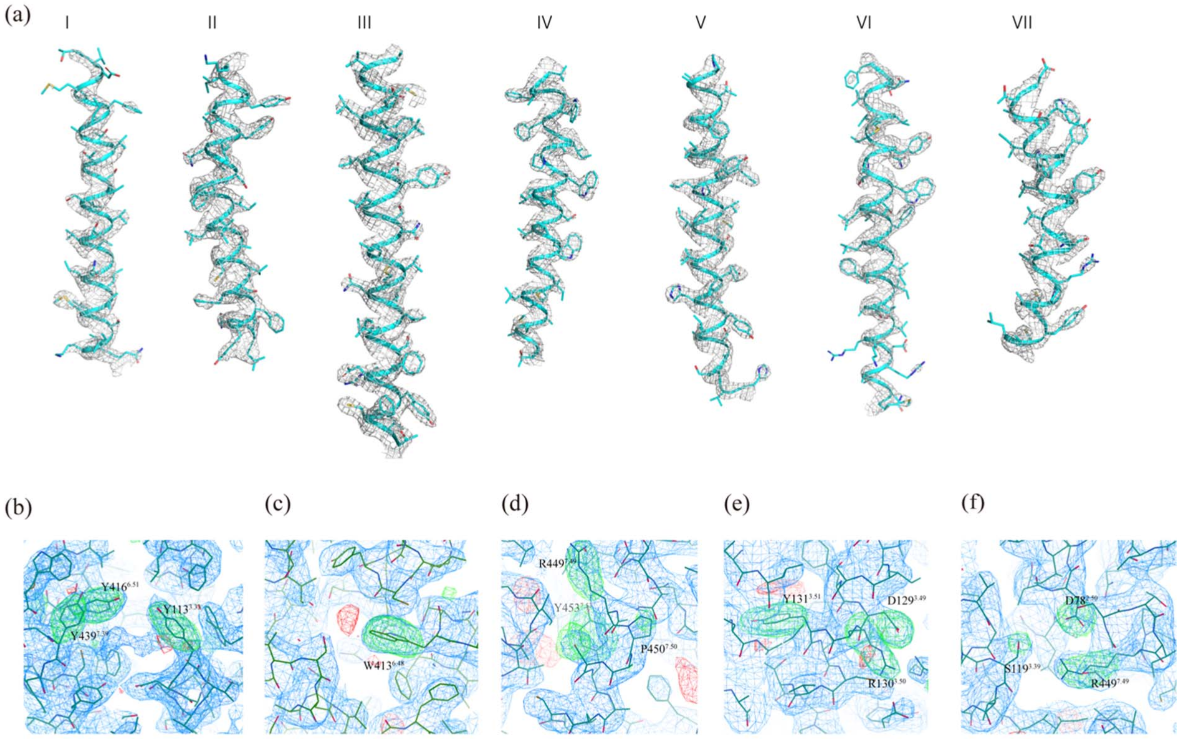

Figure S5 Omit electron density maps for the mutation-induced M4 structure. (a) The initial omit $2|F_o|$-$|F_c|$ map (grey) for seven transmembrane domains (Ⅰ, Ⅱ, Ⅲ, Ⅳ, Ⅴ, Ⅵ and Ⅶ). Contoured at 2.0σ at 3.0 Å. (b-f) The omit $2|F_o|$-$|F_c|$ (blue) and $|F_o|$-$|F_c|$ (green) maps for tyrosine lid (b), residues Trp6.48 (c), the R(R449$^{7.49}$) PxxY (Y453$^{7.53}$) motif (d), DRY motif (e), and ionic network residues in the mutation-induced M4 structure (f). The sidechains of the residues are selected for omit map generation. $2|F_o|$-$|F_c|$ omit maps contoured at 1.0σ at 3.0 Å, $|F_o|$-$|F_c|$ omit maps contoured at 3.5σ at 3.0 Å.
